# Brain glucose extraction is fixed at 10% despite twofold variability in resting cerebral blood flow in healthy humans

**DOI:** 10.1177/0271678X251400247

**Published:** 2025-12-07

**Authors:** Jennifer S Duffy, Hannah G Caldwell, Ryan L Hoiland, Connor A Howe, Kurt J Smith, Anthony R Bain, David B MacLeod, Philip N Ainslie, Travis D Gibbons

**Affiliations:** 1 School of Physical Education, Sport & Exercise Sciences, University of Otago, Dunedin, New Zealand; 2 Centre for Heart, Lung and Vascular Health, University of British Columbia – Okanagan Campus, Kelowna, BC, Canada; 3 The August Krogh Section for Human Physiology, Department of Nutrition, Exercise and Sports, University of Copenhagen, Copenhagen, Denmark; 4 Centre for Chronic Disease Prevention and Management, University of British Columbia, Kelowna, BC, Canada; 5 Department of Cellular and Physiological Sciences, Faculty of Medicine, University of British Columbia, Vancouver, BC, Canada; 6 Cerebrovascular Health Exercise and Environmental Research Sciences Laboratory, School of Exercise Science, Physical and Health Education, Faculty of Education, University of Victoria, Victoria, BC, Canada; 7 Department of Kinesiology, Faculty of Human Kinetics, University of Windsor, Windsor, ON, Canada; 8 Department of Anesthesiology, Duke University Medical Center, Durham, NC, USA; 9 Department of Biological Sciences, Northern Arizona University, Flagstaff, AZ, USA

**Keywords:** Cerebral metabolism, oxygen extraction, glucose extraction, cerebral blood flow, arteriovenous, aerobic glycolysis

## Abstract

In the resting, non-stimulated brain, metabolic demands are met exclusively by the delivery and extraction of glucose and oxygen at an ~6:1 ratio. Amongst healthy people at rest, there is marked variability in resting global cerebral blood flow (CBF) yet remarkably stable concentrations of circulating glucose and oxygen. Thus, we would expect interindividual variability in resting CBF to be inversely related to oxygen and glucose extraction, maintaining oxidative glucose metabolism. Herein, we investigated the fundamental relationship between CBF and substrate extraction in 75 healthy adults (27.3 ± 4.8 years) with resting measures of CBF and cross-brain concentrations of oxygen and glucose. We observed that the marked interindividual variability in CBF (<500 to >1200 mL/min) is inversely related to oxygen extraction (*R*^2^ = 0.85, *p* = 0.005) but not glucose extraction (*R*^2^ = 0.30, *p* = 0.273). The metabolic rates of oxygen and glucose (CMRO_2_ and CMRglc) are both directly correlated with CBF. However, there was a 1.6-fold greater slope for CMRglc-CBF, compared to CMRO_2_-CBF (*p* = 0.040). These findings indicate that the resting brain extracts more oxygen when delivery is low, maintaining stable CMRO_2_ and ATP production. Despite glucose being the primary oxidized substrate, the brain’s ability to adjust its extraction is limited, making CMRglc more dependent on delivery.

## Introduction

The brain is a unique metabolic organ owing to its disproportionate metabolic demand relative to total body mass.^1^ In the resting brain, the vast majority of the energetic demand is met by oxidative phosphorylation of glucose.^2^ Interestingly, despite remarkably stable arterial concentrations of glucose and oxygen amongst healthy humans at rest, there is striking interindividual variability in delivery that is, cerebral blood flow (CBF). In healthy adults with no known cognitive or neurological disease, resting CBF can vary by 50% between individuals.^3–5^ Thus, the question arises: How can the brain’s metabolic demands, which are met almost exclusively by substrate delivery, be achieved with such extreme differences in CBF between individuals? Given consistent levels of circulating oxygen and glucose, the only conceivable way this would be possible is for cerebral oxygen and glucose extraction to adjust to different rates of delivery.

The brain’s primary energy source of oxidative glucose metabolism is evidenced by a resting oxygen to glucose index of 5.46,^6^ nearly matching the 6:1 (oxygen:glucose) stoichiometric ratio required for oxidative phosphorylation. The ratio falling slightly below 6 indicates a small presence of aerobic glycolysis (AG; the metabolism of glucose without oxygen despite adequate oxygen availability^7^). AG is an energetically-inefficient metabolic process (~95% lower energy yield) yet plays an important role in cellular processes such as NAD^+^ homeostasis,^8^ neural development,^9^ synaptic plasticity and memory formation.^10,11^ Conversely, although the mechanisms are unclear, a loss of AG in the resting brain is associated with aging and neurodegeneration.^12,13^ Upon brain activation, such as during physical exercise^14,15^ and sensory stimulation,^2,16,17^ there is an increase in CBF alongside a decrease in oxygen extraction^18^ and no change in glucose extraction,^19^ resulting in the disproportionate uptake of glucose relative to oxygen (i.e., AG).^14,16,17^ The link between AG and CBF upon brain activation indicates that glucose and oxygen metabolism may behave differently upon variation in delivery.

The relevance of understanding how brain glucose and oxygen extraction change with delivery are paramount in the myriad of conditions where CBF is known to be reduced^20^ such as Alzheimer’s disease,^21,22^ hypertension,^23^ and ischemic stroke.^24^ Similarly, in response to probable reductions in CBF, many studies of healthy aging report stable CMRO_2_^25,26^ alongside increasing OEF, while others report decreases in CMRglc,^27–29^ which is in accordance with an age-related decline in AG.^12^ These outcomes support the notion that oxygen extraction is more responsive than glucose extraction to changes in CBF, which may also result in a decline of AG with reductions in CBF—a complementary yet inverse process with what is known to occur upon brain activation.

Here we have accumulated resting measures of CBF using duplex ultrasound alongside arterial and internal jugular bulb concentrations of oxygen and glucose in 75 healthy humans (21 females) aged 21–41 years. With this, we have examined and compared the interindividual relationships of cerebral oxygen extraction fraction (OEF) and glucose extraction fraction (GEF) with CBF and the resultant changes in glucose and oxygen metabolism relative to one another. We suggest that CBF may be a critical determinant for basal levels of CMRglc and AG due to a lack of adjustment in glucose extraction by the brain, reflecting what is well-established during brain activation.

## Methods

### Experimental model and subject details

We collected cerebral blood flow alongside arterial and internal jugular venous bulb blood sample data from the baseline measures of eight studies (three unpublished)^30–34^ in humans, all conducted at the University of British Columbia Okanagan. All studies recruited only healthy, physically-active, adults with no known cardiovascular or neurological diseases between the ages of 21–41 years old (mean 27.3 ± 4.8 years), with a total of 75 individuals (21 females). All studies were approved by the University of British Columbia Clinical Research Ethics Board and carried out in accordance with the Declaration of Helsinki and informed consent was obtained from all participants.

### Method details

#### Cross-brain blood sampling

In each study, arterial and central venous catheters were placed with the participant supine, using a sterile technique under local anesthesia, and assisted via the use of ultrasound guidance. To accurately isolate cerebral metabolism it is critical that blood sampled from the internal jugular vein comprises no contamination from extra-cerebral blood. This was done via cranial advancement (~15 cm) of the catheter, placing the catheter tip in the jugular bulb.^35^ Following this approach, it has been demonstrated to lead to >97% sampling of venous blood isolated from the brain.^36^ Blood samples from the artery (brachial or radial) and internal jugular vein were drawn simultaneously and immediately analyzed using a commercial blood gas analyzer (ABL90 FLEX Radiometer).

#### Cerebral blood flow (CBF)

Simultaneous diameter and velocity of the right internal carotid artery (ICA) and left vertebral artery (VA) were measured using 10 MHz multi-frequency linear array duplex ultrasound (Terasmart uSMart 3300, Teratech, Burlington, MA, USA). Simultaneous B-mode imaging and pulse-wave mode were used to measure live arterial diameter and beat-by-beat peak velocity respectively.

Cerebral artery diameter and blood velocity were analyzed with custom wall tracking/edge detection software (BloodFlow Analysis, version 5.1). Volumetric blood flow (*Q*) was calculated from blood flow analysis data via equations (1) and (2).

### Quantification and statistical analysis

(1) $$\begin{array}{l} QICA\;or\;QVA\;\left(ml/min\right)\;=\;\\ \left(0.5\;x\;peak\;envelope\;velocity\right)\;x\;\\ \left(\pi {\left(0.5\;x\;diameter\right)}^{2}\right)\;x\;60 \end{array}$$

Global cerebral blood flow was calculated as twice the sum of unilateral assessments of ICA and VA blood flow:

(2) $$\begin{array}{l} Global\;cerebral\;blood\;flow\;\left(mL/min\right)\;=\;\\ 2\;x\;\left(QICA\;x\;QVA\right) \end{array}$$

*Blood oxygen content (mL/dL):*

(3) $$C_{a}O_{2}and\;C_{v}O_{2}=\;\left[Hb\right]\;x\;SO_{2}+\;0.003\;x\;PO_{2},$$

where arterial and venous oxygen content (C_a_O_2_ and C_v_O_2_) quantifies the total amount of oxygen in the blood such that [Hb] represents hemoglobin concentration, SO_2_ oxygen saturation and PO_2_ partial pressure of oxygen.

*Extraction Fraction (%)*

(4) $$\begin{array}{l} Oxygen\;extraction\;fraction\;\left(OEF\right)\;=\\ \left(\left(C_{a}O_{2}-\;C_{V}O_{2}\right)/C_{a}O_{2}\right)\;x\;100 \end{array}$$

(5) $$\begin{array}{l} Glucose\;extraction\;fraction\;\left(GEF\right)\;=\;\;\\ \left(\left(C_{a}glc\;-\;C_{v}glc\right)/C_{a}glc\right)\;x\;100, \end{array}$$

where C_a_glc and C_v_glc represent the arterial and venous concentrations of glucose in mmol/L.

*Cerebral Metabolic Rate*

(6) $$CMRO_{2}\left(mL/min\right)\;=\;\left(CBF\;/\;100\right)\;x\;\left(C_{a}O_{2}-\;C_{v}O_{2}\right)$$

(7) $$\begin{array}{l} CMRglc\;\left(mmol/min\right)\;=\;\\ \left(CBF/1000\right)\;x\;\left(C_{a}glc\;-\;C_{v}glc\right) \end{array}$$

*Aerobic glycolysis (AG)*

(8) $$AG=\;CMRglc\;-\;CMRO_{2}/6$$

Here, oxygen content is converted to mmol/L for the calculation of CMRO_2_ and divided by 6 to account for the 1:6 stoichiometric ratio of glucose:oxygen. AG is defined as the metabolism of glucose without oxygen despite adequate oxygen availability.^7,37^ Thus, the calculated value for AG provides the rate of excess glucose extracted by the brain in mmol/min relative to what can be accounted for by oxidative phosphorylation. A greater positive value indicates greater rates of AG while a negative value indicates unaccounted for sources of carbohydrates (e.g., lactate, ketones, and brain glycogen) undergoing oxidation.

### Statistical analysis

All relationships were determined by a linear mixed effects model in R (R Statistical Software v4.4.1; R Core Team 2024). P_a_CO_2_, study, and sex were included as covariates to account for the direct effects of P_a_CO_2_ on CBF, the effect of brain size between males and females, and any effect of differences in experimenter techniques or standardizations between studies. Fourteen participants were involved in two or more studies (at least 1 year apart), thus subject identifiers were used as random intercepts. The variance inflation factor (VIF) was used to assess the effect of collinearity between variables such that a VIF less than 2 was deemed acceptable and suggesting in minimal effects on model estimates. Significance was set at *p* < 0.05. Outliers were removed if the oxygen to glucose ratio was 2 standard deviations outside the mean as these values likely reflect experimental error or brain activation outside the normal resting brain. The equation of the line, *p*-value, and conditional *R*^2^ value are indicated in the figure.

## Results

### Oxygen extraction inversely changes with variation in CBF, glucose extraction does not

**Figure 1.**
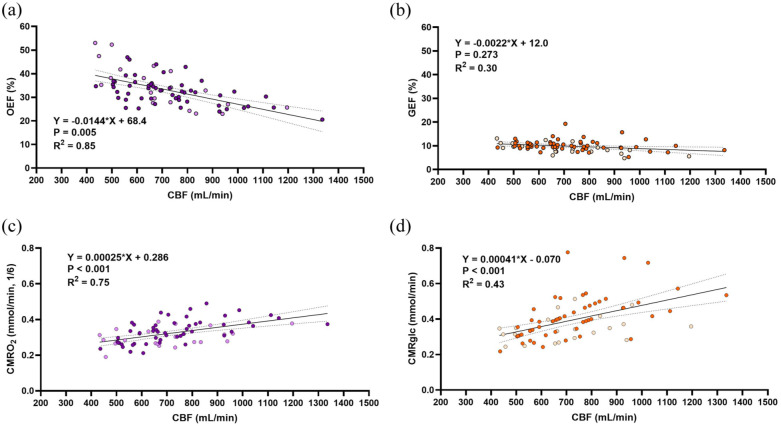
Oxygen extraction inversely changes with variation in CBF, glucose extraction does not. (a) Depicts the OEF-CBF relationship such that there is a significant inverse relationship between. (b) Depicts the GEF-CBF relationship such that there is a non-significant inverse relationship and a 6.5-fold lower slope than that of OEF-CBF (*p* < 0.001). Panel (c) and (d) depict the regression for CMRO_2_-CBF and CMRglc-CBF, respectively, both significantly correlated with CBF. CMRO_2_ is converted to mmol/min and divided by 6 to facilitate comparison of proportional changes in metabolism with glucose based on stoichiometry. This results in a 1.6-fold greater change in CMRglc relative to CMRO_2_ for a given change in CBF. In all figures, *n* = 75 and females are indicated by lightly colored data points. All results were determined by a linear mixed effects model with PaCO_2_, study, and sex as fixed effects and subject identifiers as random effects. Equations of the line and statistics are displayed in each respective panel after accounting for these factors, while raw data and simple linear regressions depict the trend on each graph. Significance was set at *p* < 0.05, dotted lines represent the 95% confidence interval.

In 75 resting healthy adult subjects (age 21–41 years, mean 27.3 ± 4.8 years), we present striking interindividual variability in CBF with the lower 10th percentile having CBF of <500 mL/min, whereas the upper 10th percentile have a CBF of >1000 mL/min. Additionally, OEF is on average 3.4-fold larger than GEF, with average values of 33.1 ± 6.8% and 9.8 ± 2.3%, respectfully. We then examined the relationships between oxygen and glucose extraction fractions for a given CBF by plotting each subject’s OEF and GEF with their respective CBF (Figure 1(a) and (b)). We show that OEF decreases 1.4% for every dL/min increase in CBF (*R*^2^ = 0.85, *p* = 0.005), while the slope of the GEF-CBF relationship is not different from zero (*p* = 0.273). Moreover, the OEF-CBF slope is 6.5-fold larger than GEF-CBF, thus the difference is unaccounted for by the 3.4 greater magnitude, demonstrating the greater dependence of OEF on CBF. Therefore, unlike for oxygen, there is no compensation in glucose extraction with vastly different resting values of CBF.

**Figure 2.**
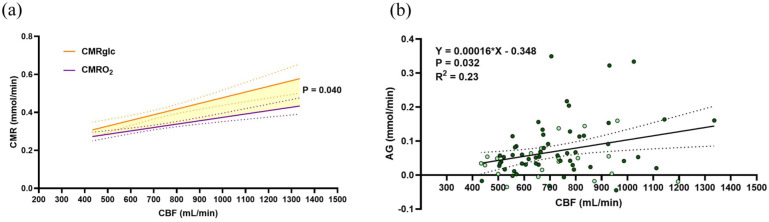
Inability of glucose extraction to accommodate low CBF underlies low rates of AG. (a) Depicts both the relationship of CMRglc and CMRO_2_ on the same axis, with a significant difference between slopes. (b) Depicts the AG-CBF relationship which indicates that with an increase in CBF there is an increase in excess glucose metabolism relative to oxygen. In both figures, *n* = 75 and females are indicated by lightly colored data points. All results were determined by a linear mixed effects model with PaCO_2_, study, and sex as fixed effects and subject identifiers as random effects. Equations of the line and statistics are displayed in each respective panel after accounting for these factors, while raw data and simple linear regressions depict the trend on each graph. Significance was set at *p* < 0.05, dotted lines represent the 95% confidence interval. Comparison of slopes was determined via the interaction effect of metabolic variable (CMRglc & CMRO_2_) and CBF.

We then determined the relationship between the metabolism of oxygen and glucose (CMRO_2_ and CMRglc) with the variability in CBF (Figure 1(c) and (d)). CMRO_2_ is scaled to it’s stoichiometric ratio for glucose (divided by 6) to account for the greater oxygen extraction required to facilitate oxidative glucose metabolism. Both relationships yield a significant, positive slope, suggesting that the significant changes in OEF do not fully accommodate differences in CBF, that is, a perfectly accommodating extraction fraction would yield stable CMRO_2_. However, upon comparing the slopes of the CMRO_2_-CBF and CMRglc-CBF relationships (Figure 2(a)), we revealed that CMRglc is 1.6 times more dependent on CBF compared to CMRO_2_ (slopes 0.00041 vs 0.00025, *p* = 0.040). This reflects a stronger association between glucose metabolism and delivery compared to oxygen.

Variance inflation factors (VIF) were established to assess effects of multicollinearity on the previous regressions due to CBF being a component in the calculation of both CMRO_2_ and CMRglc. All values were below 2, indicating an acceptable level of collinearity that is unlikely to significantly impact model estimates.

### Inability of glucose extraction to accommodate low CBF predicts low rates of AG

To extend on the finding that CMRglc varies more with interindividual differences in CBF (Figure 2(a)) we quantified individual resting levels of aerobic glycolysis according to equation (8) (see Methods for details on calculation and interpretation). A linear regression of AG and CBF (Figure 2(b)) resulted in a positive slope (*p* = 0.032) corroborating the disproportionally greater increase in CMRglc relative to CMRO_2_ amongst individuals with greater CBF. This relationship also predicts that AG will be 0 mmol/min when CBF is 292 mL/min or less.

This analysis was repeated using normalized CBF (nCBF) expressed in terms of 100 g of brain mass. The average brain volume based on age and sex reported in a recent metanalysis^38^ was used to estimate brain mass for each individual.^39^ The overall outcomes were the same, demonstrating that brain mass does not explain the discrepancy between oxygen and glucose extraction across the wide range of CBF (see Supplemental Material).

## Discussion

We have demonstrated that brain glucose extraction does not adapt to striking variability in resting CBF, while oxygen extraction does. Cerebral metabolism is derived from the product of the rate of substrate delivery and the arterial-venous difference (i.e., extraction).^40,41^ As such, the way substrate extraction responds (or does not respond) to changes in delivery will determine the metabolic rate. Thus, the greater dependence of glucose metabolism on delivery suggests that low CBF places greater strain on CMRglc compared to CMRO_2_. This inability of the brain to adjust glucose extraction underlies the observation that low CBF is directly related to low rates of AG. The inverse relationship of OEF with oxygen delivery is a well-established phenomena in the dynamic response to brain activation^17,42^ (providing the fundamental basis for BOLD-MRI), yet is completely unexplored in the resting brain across individuals where CBF varies substantially.

A possible explanation for the broader range of compensable oxygen metabolism relative to glucose is the difference in oxygen and glucose transport across the blood brain barrier and into cells. Oxygen follows passive diffusion which depends on oxygen tension gradients between blood and tissue,^43^ while glucose influx requires facilitated diffusion through GLUT1/GLUT3 transporters,^44,45^ which can be influenced by transporter saturation and expression levels. Therefore, while CBF and extraction may covary differently for each substrate, the disproportionate rates of glucose and oxygen metabolism at either extreme of delivery could reflect the differing mechanisms of their transport. Additionally, an increase in substrate delivery can occur via increases in circulating concentrations. Recent work by Blazey et al., whereby circulating glucose was elevated to 17 mmol/L, showed an increase in CMRglc but not CMRO_2_, that is, elevated AG, in white matter with no change in CBF.^46^ Thus, it appears that in the healthy brain CMRglc is somewhat dependent on glucose delivery, whether due to alterations in flow (CBF) or circulating glucose concentration. This is in stark contrast to CMRO_2_, which is stable during hyperglycemia,^46^ hypoglycemia,^47^ hypoxia and hyperoxia.^48^ Although we cannot discern cause-effect with our data, the disparate relationships between glucose and oxygen delivery and extraction fraction help explain the relationship between AG and cerebral glucose and oxygen delivery.

The understanding that with age and in several pathological states (e.g., Alzheimer’s disease, ischemic stroke, and cognitive impairment) humans experience a decrease in CBF^20,49,50^ as well as glucose hypometabolism^21,22^ suggests that these two variables are inextricably linked. This link poses a ‘chicken or the egg’ dilemma: Does a decrease in CBF facilitate brain glucose hypometabolism or is reduced CBF a reflection of lower metabolic demand? Given the finding that the age-related reduction in cerebral glucose metabolism is driven almost entirely by a reduction in AG^12^—a non-oxidative and *low energy-yielding process*—we posit that the former presents a more teleologically sensible explanation. Conversely, if the higher glucose metabolism that is associated with higher CBF was to be accompanied by oxygen metabolism, then one may conclude this relationship is instead driven by energetic demands.

When considering these data and the proposed link between substrate delivery and metabolism in healthy human adults at rest, it is important to consider what may be driving the marked variability in CBF. As discussed, cerebral activation results in an increase in CBF due to reasons such as the removal of protons and maintenance of partial pressures of carbon dioxide and oxygen.^18^ An increase in CBF during activation may also be a response to an increased demand for glucose.^51^ While all measures presented here were taken under quiet, resting conditions, it is possible that confounding brain activation is present in some of these individuals, driving the variability in CBF and AG. Moreover, arterial levels of carbon dioxide (PaCO_2_), are an important determinant of CBF during wakefulness.^52,53^ Likewise, studies have attributed variations in OEF to differences in PaCO_2_, both at rest^54^ and under experimentally induced changes.^55^ As such, PaCO_2_ was included as a covariate to account for subject differences in arterial CO_2_ levels.

Overall, interindividual variability in CBF is accompanied by variability in oxygen but not glucose extraction, and this underlies the tight coupling between CBF and non-oxidative glucose metabolism, i.e., aerobic glycolysis. This can be attributed to a smaller change in oxygen metabolism for a given CBF relative to glucose metabolism, an outcome which is well-established during intraindividual brain activation but undescribed in interindividual CBF variability. Future work should aim to examine this relationship in aging and pathological reductions of CBF to establish underlying reasons for reductions in glucose metabolism and AG.

## Supplemental Material

sj-csv-2-jcb-10.1177_0271678X251400247 – Supplemental material for Brain glucose extraction is fixed at 10% despite twofold variability in resting cerebral blood flow in healthy humans Supplemental material, sj-csv-2-jcb-10.1177_0271678X251400247 for Brain glucose extraction is fixed at 10% despite twofold variability in resting cerebral blood flow in healthy humans by Jennifer S Duffy, Hannah G Caldwell, Ryan L Hoiland, Connor A Howe, Kurt J Smith, Anthony R Bain, David B MacLeod, Philip N Ainslie and Travis D Gibbons in Journal of Cerebral Blood Flow & Metabolism

sj-docx-1-jcb-10.1177_0271678X251400247 – Supplemental material for Brain glucose extraction is fixed at 10% despite twofold variability in resting cerebral blood flow in healthy humans Supplemental material, sj-docx-1-jcb-10.1177_0271678X251400247 for Brain glucose extraction is fixed at 10% despite twofold variability in resting cerebral blood flow in healthy humans by Jennifer S Duffy, Hannah G Caldwell, Ryan L Hoiland, Connor A Howe, Kurt J Smith, Anthony R Bain, David B MacLeod, Philip N Ainslie and Travis D Gibbons in Journal of Cerebral Blood Flow & Metabolism
