# Supplementary material for: Brain glucose extraction is fixed at 10% despite twofold variability in resting cerebral blood flow in healthy humans
Source: J Cereb Blood Flow Metab. 2025 Dec 7:0271678X251400247. Online ahead of print. doi: 10.1177/0271678X251400247 (PMC12685695; doi:10.1177/0271678X251400247)
Supplement: sj-docx-1-jcb-10.1177_0271678X251400247 – Supplemental material for Brain glucose extraction is fixed at 10% despite twofold variability in resting cerebral blood flow in healthy humans [file sj-docx-1-jcb-10.1177_0271678X251400247.docx]

**Supplemental Material**

**Brain glucose extraction is fixed at 10% despite twofold variability in resting cerebral blood flow in healthy humans**

**Figure S1. Oxygen extraction inversely changes with variation in normalized CBF (nCBF), glucose extraction does not.** (A) depicts the OEF-nCBF relationship such that there is a significant inverse relationship. (B) depicts the GEF-nCBF relationship such that there is a non-significant inverse relationship and a 5.9-fold lower slope than that of OEF-nCBF (p<0.0001). Panel C and D depict the regression for nCMRO_2_-nCBF and nCMRglc-nCBF, respectively, both significantly correlated with nCBF. nCMRO_2_ is converted to mmol/min and divided by 6 to facilitate comparison of proportional changes in metabolism with glucose based on stoichiometry. This results in a 1.6-fold greater change in nCMRglc relative to nCMRO_2_ for a given change in nCBF. In all figures, n=75 and females are indicated by lightly coloured data points. All results were determined by a linear mixed effects model with PaCO_2_, study, and sex as fixed effects and subject identifiers as random effects. Equations of the line and statistics are displayed in each respective panel after accounting for these factors, while raw data and simple linear regressions depict the trend on each graph. Significance was set at P<0.05, dotted lines represent the 95% confidence interval. These outcomes simply extend the main findings with values scaled to brain mass.

**Figure S2. A greater change in glucose metabolism with nCBF results in variation in resting aerobic glycolysis.** (A) depicts both the relationship of nCMRglc and nCMRO_2_ on the same axis, with a significant difference between slopes. (B) depicts the nAG-nCBF relationship which indicates that with an increase in nCBF there is an increase in excess glucose metabolism relative to oxygen. In both figures, n=75 and females are indicated by lightly coloured data points. All results were determined by a linear mixed effects model with PaCO2, study, and sex as fixed effects and subject identifiers as random effects. Equations of the line and statistics are displayed in each respective panel after accounting for these factors, while raw data and simple linear regressions depict the trend on each graph. Significance was set at P<0.05, dotted lines represent the 95% confidence interval. Comparison of slopes was determined via the interaction effect of metabolic variable (nCMRglc & nCMRO_2_) and nCBF. These outcomes simply extend the main findings with values scaled to brain mass.
